# The impact of HIV on children's education in eastern Zimbabwe

**DOI:** 10.1080/09540121.2014.892564

**Published:** 2014-03-14

**Authors:** Erica L. Pufall, Constance Nyamukapa, Jeffrey W. Eaton, Catherine Campbell, Morten Skovdal, Shungu Munyati, Laura Robertson, Simon Gregson

**Affiliations:** a Department of Infectious Disease Epidemiology, Imperial College London, London, UK; b Biomedical Research and Training Institute, Harare, Zimbabwe; c London School of Economics and Political Science, Institute of Social Psychology, London, UK; d Department of Public Health, University of Copenhagen, Copenhagen, Denmark

**Keywords:** HIV, children, education, orphanhood, Zimbabwe

## Abstract

Little is known about how HIV impacts directly and indirectly on receiving, or particularly succeeding in, education in sub-Saharan Africa. To address this gap, we used multivariable logistic regression to determine the correlation between education outcomes in youth (aged 15–24) (being in the correct grade-for-age, primary school completion and having at least five “O” level passes) and being HIV-positive; having an HIV-positive parent; being a young carer; or being a maternal, paternal or double orphan, in five rounds (1998–2011) of a general population survey from eastern Zimbabwe. The fifth survey round (2009–2011) included data on children aged 6–17, which were analysed for the impacts of the above risk factors on regular attendance in primary and secondary schools and being in the correct grade-for-age. For data pooled over all rounds, being HIV-positive had no association with primary school completion, “O” level passes, or being in the correct grade-for-age in adolescents aged 16–17 years. Additionally, HIV status had no significant association with any education outcomes in children aged 6–17 surveyed in 2009–2011. In 2009–2011, being a young carer was associated with lower attendance in secondary school (69% vs. 85%, AOR: 0.44; *p* = 0.02), whilst being a maternal (75% vs. 83%, AOR: 0.67; *p* < 0.01), paternal (76% vs. 83%, AOR: 0.67; *p* = 0.02) or double (75% vs. 83%, AOR: 0.68; *p* = 0.02) orphan was associated with decreased odds of being in the correct grade-for-age. All forms of orphanhood also significantly decreased the odds of primary school completion in youths surveyed from 1998 to 2011 (all *p* < 0.01). We found no evidence that HIV status affects education but further evidence that orphans do experience worse education outcomes than other children. Combination approaches that provide incentives for children to attend school and equip schools with tools to support vulnerable children may be most effective in improving education outcomes and should be developed and evaluated.

## Introduction

In sub-Saharan Africa (SSA), the impact of the HIV epidemic on children's education is only now beginning to be properly understood. The majority of children affected by HIV are of school-going age and live in countries where education is not compulsory and school fees exist (Boerma, Urassa, Senkoro, Klokke, & Ngẃeshemi, 1999; UNAIDS, UNICEF, & USAID, 2004). School fees are particularly problematic for children from households impacted by HIV due to lost income from sick or deceased adult relatives and the high costs of medicines (Fenner et al., 2010). In addition to the financial barriers, children may stop attending school because they have to care for sick family members, or may themselves be HIV-positive. Indeed, in the Central African Republic and Swaziland, AIDS caused school enrolment to fall by 25–30% at the beginning of the millennium (UNAIDS, 2002) and, in a high-density community in Zimbabwe, nearly 72% of children affected by AIDS were not in school, compared to just 29% of children not affected by AIDS (Kembo, 2010).

On an individual level, it has been shown that a child's education can be negatively impacted by either the loss of a parent or having an HIV-positive parent. Loss of a father may result in children dropping out of school (Case, Paxson, & Ableidinger, 2004), while maternal death has been associated with children not enrolling in school, delaying school attendance, lower educational attainment, being at a lower grade for their age and worse performance in school (Ainsworth, Beegle, & Koda, 2005; Birdthistle et al., 2009; Case & Ardington, 2006; Evans & Miguel, 2007; Nyamukapa & Gregson, 2005). Children with sick parents have also been shown to be at a lower grade for their age due to interruptions caused by looking after their ailing parent (s) (Ainsworth et al., 2005; Kasirye & Hisali, 2010).

An increasing number of studies have examined the effects of parental loss and illness on education outcomes in SSA. A recent review noted that, of the 23 peer-reviewed studies on the effects of HIV/AIDS on children's education outcomes published between 1999 and 2010, none had directly examined the effects of children being HIV-positive themselves (Guo, Li, & Sherr, 2012). AIDS-related illness experienced by HIV-positive children and adolescents could be expected to impact negatively on attendance and, therefore, on grade progression (Bandason et al., 2013). Moreover, research suggests that perinatal HIV infection can cause developmental delay and impact cognitive development at a young age (Mayaux et al., 1996; McDonald et al., 2012; Puthanakit et al., 2013; Smith et al., 2000), which could have negative consequences for school performance in both primary and secondary school children.

Although there is a general consensus that HIV and its downstream impacts have negative consequences for education outcomes, the type and magnitude of those effects can vary dramatically by country. Differences between countries in traditional customs and socio-cultural, economic, policy and political situations influence the barriers and opportunities for education among vulnerable children (Atekyereza, 2001; Guo et al., 2012; Nyamukapa, Foster, & Gregson, 2003; Urassa et al., 1997). The effect of orphanhood on enrolment and attendance varies with age, gender, religion and household composition (Ainsworth et al., 2005; Ainsworth & Filmer, 2006), all potential cofactors in the relationship between orphanhood and school attendance/enrolment (Birdthistle et al., 2009; Kürzinger et al., 2008; Sharma, 2006).

In SSA, Zimbabwe provides an excellent setting in which to study education outcomes because, although primary school fees were introduced in 1991 and have risen steadily since (Kanyongo, 2005), literacy rates remain among the highest in Africa, with education being a clear priority in many families. We investigated the impact of a range of HIV-related effects on children's and adolescents’ education outcomes in the Manicaland province of Zimbabwe, using pooled data for adolescents from five survey rounds conducted from 1998 to 2011 and data for children from a dedicated child survey (2009–2011). Dimensions of vulnerability to HIV investigated were (1) being HIV-positive; (2) having an HIV-positive or AIDS-sick parent; (3) being a maternal, paternal orphan or double orphan; and 4) being a young carer. High school enrolment in the study population (over 90% in 2009–2011) allowed us to examine the effects of HIV not only on school attendance but also on quality of education. Education outcomes considered were (1) regular attendance of primary or secondary school, (2) enrolment in the correct grade for age, (3) completion of primary school, and (4) having acquired at least five “O” level passes (final year examinations, of which five passes are the minimum requirement for formal sector employment).

## Methods

### Study population and data collection

The Manicaland HIV/STD Prevention Project is a population-based, open- cohort survey in the Manicaland province of eastern Zimbabwe. Five rounds of the survey were conducted between 1998 and 2011. Each round involves a census of all households in the 12 study sites (2 small towns; 2 roadside settlements; 4 subsistence farming areas; and 4 large-scale agricultural estates), followed by random sampling of individual household members aged 15–54 for interview, with roughly 10,000 individuals interviewed in each round. As part of the most recent round (2009–2011), a child survey component was added. A total of 5520 two- to seventeen-year-olds were interviewed from a random sample of households using a structured questionnaire similar to that employed in the adult survey. Dried blood spot samples were taken from all participants and tested for HIV in an offsite laboratory using the COM-BAIDS-RS HIV 1 + 2 Immunodot Assay (Span Diagnostics, India); HIV positives were confirmed using Vironostika HIV Uni-form II Plus O (Biomérieux, France). Ethical approval for the Manicaland HIV/STD Prevention Project was obtained from the Medical Research Council of Zimbabwe, the Biomedical Research and Training Institute Zimbabwe's institutional review board, and the Imperial College London Research Ethics Committee. Written informed consent was obtained prior to survey participation from the participant or their primary carer, if under the age of 18. In addition, children aged 7–12 and adolescents aged 13–17 years provided verbal or written assent, respectively. Participants and guardians were informed that, at any point, they could refuse to answer a question or decline to continue the interview.

### Education variables

Educational outcomes were measured using different variables in the adult and child surveys. From the adult survey, data were pooled over all rounds and educational outcomes were completion of primary school (aged 15–24), having at least five “O” level passes (aged 16–24) and being in the correct grade-for-age (aged 15–20). Grade-for-age was used to measure progress in schooling and adolescents were deemed to be progressing normally if they were no more than two years behind in school (Miller, 2006). The two-year leeway allowed for the differing ages when children start school.

For the child survey, education outcomes were primary school completion (aged 13–17), being in the correct grade-for-age (aged 8–17) and regular attendance for both primary (aged 6–12) and secondary (aged 13–17) school. Regular attendance was defined as having attended at least 80% of the last 20 school days. Children who were not enrolled in school were included in the analysis as children who did not attend regularly.

### Data analysis

Logistic regression was used to determine associations between the education outcomes and children made vulnerable by AIDS, either through being HIV-positive; being a young-carer; having an HIV-positive parent; or being a maternal, paternal or double orphan. Comparison groups were individuals who were unaffected by HIV/AIDS. Models were adjusted for age (linearly, by single year of age), gender, socio-economic status (measured using a previously described index (Lopman et al., 2007)), community type (subsistence farming, roadside settlement, town or estate) and round of data collection (if applicable). Individual random effects were included to account for repeated measures of the same individuals in multiple survey rounds and household random effects were included to account for residual correlation between children residing in the same household. Analysis of the association between being HIV-positive and education outcomes for young adults included only persons aged 15–17 in rounds 4 and 5, of whom, HIV-positive persons were most likely long-term survivors of mother-to-child transmission (MTCT) (Eaton et al., 2013), to avoid the reverse causality that poor education outcomes contributed to subsequent risk of HIV infection. In cases where orphanhood was significantly associated with an education outcome, we tested whether age of orphanhood had an impact on that outcome.

## Results

Table 1 presents characteristics of vulnerable and non-vulnerable children in 2009–2011 (round five of the survey). There were no significant differences between the different types of vulnerable children, except for young carers being older than other children (mean age: 14.4 years vs. 10.8 years; *p* < 0.01). All categories of vulnerable children were more likely to come from households receiving external assistance than non-vulnerable children (all *p* values < 0.05). Additionally, young carers were significantly more likely to come from households receiving schooling support than other categories of children (14.0% vs. 3.6%, *p* < 0.01). When comparing children receiving external support to those not, although the numbers are small, orphans from households receiving support for schooling (*n* = 91) were significantly more likely (76.7% vs. 50.0%, *p* = 0.03) to be in the correct grade-for-age than orphans not receiving support.

**Table 1.** Characteristics of vulnerable and non-vulnerable children (aged 6–17) in round five (2009–2011) of the Manicaland survey.

	Non-vulnerable (*n*: 2518, 55.0%)	HIV+ child (*n:* 94, 2.1%)	HIV+ parent (not ill) (*n:* 585, 12.8%)	HIV+ parent (ill) (*n:* 178, 3.9%)	Young carer (*n:* 86, 1.9%)	Maternal orphan (*n:* 727, 15.9%)	Paternal orphan (*n:* 1406, 30.7%)	Double orphan (*n:* 483, 10.6%)
Average age	12.4	12.5	12.7	12.8	14.4	13.6	13.4	13.9
Percent male	49.3%	53.2%	48.6%	49.4%	41.9%	48.8%	49.6%	50.1%
*Site type*								
Town	13.9%	18.1%	17.6%	19.1%	15.1%	18.0%	15.7%	17.2%
Commercial estate	23.9%	11.7%	25.0%	22.5%	27.9%	17.1%	18.4%	15.9%
Subsistence farming	42.4%	50%	35.6%	36.0%	39.5%	39.3%	42.0%	42.7%
Roadside trading centre	19.8%	20.2%	21.9%	22.5%	17.4%	25.6%	23.8%	24.2%
Average SES	0.33	0.33	0.31	0.30	0.29	0.31	0.29	0.30
*Percent receiving external support*								
Any form of support	10.5%	17.0%	15.2%	16.3%	23.3%	14.4%	14.4%	14.7%
Education support	1.1%	4.3%	4.4%	7.3%	14.0%	4.4%	4.6%	4.6%

*Note:* Percentages of vulnerable and non-vulnerable children do not add up to 100% as some children are in more than one category of vulnerability.

**Figure 1.**
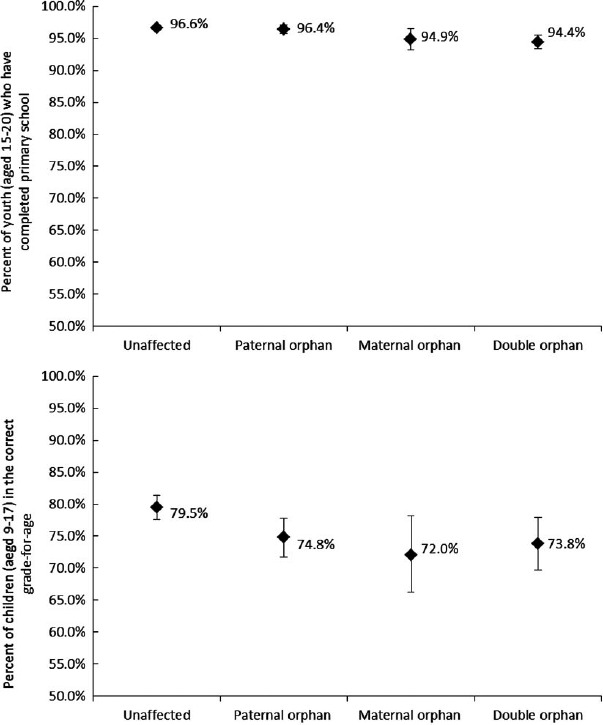
Unadjusted differences in percentage of children who have completed primary school (1a) and who are in the correct grade for age (1b) as a function of orphanhood status, compared to children unaffected by the HIV-epidemic.

Being HIV-positive was not significantly associated with any education measures in youth aged 15–17 years pooled from rounds 4 and 5 (Table 2) or in children aged 6–17 surveyed from 2009 to 2011 (Table 3). Young carers were less likely to attend secondary school (69.0% vs. 85.2%, AOR: 0.44, *p* = 0.02) from 2009 to 2011, a difference that was seen in both male and female young carers. However, being a young carer had no association with primary school attendance. Maternal (74.9% vs. 82.6%, AOR: 0.67, *p* < 0.01), paternal (76.3% vs. 82.6%, AOR: 0.67, *p* = 0.02) and double (75.0% vs. 80.6%, AOR: 0.68, *p* = 0.02) orphans were all less likely to be in the correct grade-for-age amongst children aged 8–17 years surveyed from 2009 to 2011. Youth aged 15–20 years pooled over all rounds were less likely to have completed primary school if they were maternal (94.6% vs. 96.7%, AOR: 0.44, *p* < 0.01), paternal (95.7% vs. 96.8%, AOR: 0.59, *p* < 0.01) or double (94.4% vs. 96.4%, AOR: 0.47, *p* < 0.01) orphans. However, the effect of orphanhood was not cumulative: double orphans were no more likely than maternal or paternal orphans to suffer educational setbacks (Figure 1) after adjusting for covariates, and orphanhood was not associated with primary or secondary school attendance or with “O” level passes. Age at orphanhood was not significantly associated with any of the education outcomes.

**Table 2.** Associations between measures of childhood vulnerability and education outcomes in data from youth (aged 15–24 years) pooled over five survey rounds (1998–2011).

	Correct grade for age (ages 15–20)			Completed primary (ages 15–24)			5+ O level passes (ages 16–24)		
	*n/N* (%)	AOR (95% CI)	*p*	*n/N* (%)	AOR (95% CI)	*p*	*n/N* (%)	AOR (95% CI)	*p*
HIV+^a^	1177/1370 (85.9%)	0.87 (0.32–2.35)	0.78	1674/1754 (95.4%)	0.75 (0.12–4.56)	0.75	70/1,596 (4.4%)	0.48 (0.12–2.00)	0.32
*HIV*+ *parent*									
Parent not ill	892/1260 (70.8%)	1.23 (0.90–1.70)	0.20	1346/2137 (96.3%)	1.16 (0.55–2.44)	0.70	168/1,729 (9.7%)	1.45 (0.91–2.33)	0.12
Parent ill	946/1356 (69.8%)	0.88 (0.60–1.28)	0.67	2405/2505 (96.0%)	1.03 (0.57–1.86)	0.93	217/2,089 (10.4%)	1.42 (0.80–2.50)	0.23
Young carer	1448/2184 (66.3%)	1.25 (0.95–1.64)	0.10	7858/8160 (96.3%)	0.92 (0.59–1.44)	0.71	795/5,519 (14.4%)	1.11 (0.85–1.45)	0.46
*Orphanhood*^b^									
Maternal orphan	2483/3641 (68.2%)	0.83 (0.65–1.06)	0.14	6097/6351 (96.0%)	0.44 (0.29–0.67)	<0.01	418/5,468 (7.6%)	0.93 (0.66–1.31)	0.68
Paternal orphan	3155/4586 (68.8%)	0.99 (0.81–1.19)	0.90	8705/9042 (96.3%)	0.59 (0.40–0.86)	<0.01	522/6,914 (7.5%)	1.00 (0.74–1.34)	0.98
Double orphan	3034/4429 (68.5%)	0.85 (0.67–1.10)	0.22	7701/8015 (96.1%)	0.47 (0.31–0.71)	<0.01	510/6,740 (7.6%)	0.90 (0.62–1.32)	0.60

Note: Odds ratios are adjusted for age, sex, round, SES and site type, with household and individual (to account for repeat measurements) random effects.

*n/N*: number of youth who have attained the education outcome/total number of youth included in each analysis (**NB**: *N* differs for each analysis due to different response rates for the questions on the vulnerability categories).

a Ages 15–17, rounds 4 and 5 only.

b Ages 15–20.

**Table 3.** Associations between measures of childhood vulnerability and education outcomes in children in most recent survey round (2009–2011).

	Correct grade for age (ages 8–17)			Primary attendance (ages 6–12)			Secondary attendance (ages 13–17)		
	*n/N* (%)	AOR (95% CI)	*p*	*n/N* (%)	AOR (95% CI)	*p*	*n/N* (%)	AOR (95% CI)	*p*
HIV+	1632/1995 (81.8%)	0.77 (0.38–1.56)	0.47	1115/1216 (91.7%)	0.88 (0.14–5.53)	0.89	481/729 (66.0%)	1.67 (0.43–6.48)	0.46
*HIV*+ *parent*									
Parent not ill	544/684 (79.5%)	1.19 (0.73–1.93)	0.49		N/A^a^		184/259 (71.0%)	1.04 (0.56–1.95)	0.89
Parent ill	488/645 (75.6%)	0.75 (0.43–1.31)	0.32	173/194 (89.2%)	0.47 (0.05–4.73)	0.52	177/257 (68.9%)	1.23 (0.55–2.76)	0.61
Young carer	1044/1265 (82.5%)	0.83 (0.43–1.59)	0.57	1096/1195 (91.7%)	0.73 (0.06–9.56)	0.81	519/623 (83.3%)	0.47 (0.25–0.89)	0.02
*Orphanhood*									
Maternal orphan	1979/2453 (80.7%)	0.67 (0.51–0.88)	<0.01	1299/1409 (92.2%)	1.59 (0.53–4.75)	0.41	637/922 (69.1%)	0.77 (0.52–1.12)	0.17
Paternal orphan	2377/2967 (80.1%)	0.67 (0.60–0.96)	0.02	1514/1644 (92.1%)	1.16 (0.57–2.36)	0.68	769/1114 (69.0%)	0.80 (0.57–1.12)	0.19
Double orphan	2306/2890 (79.8%)	0.68 (0.49–0.93)	0.02	1487/1613 (92.2%)	1.63 (0.50–5.31)	0.42	744/1088 (68.4%)	0.73 (0.46–1.16)	0.18

Note: Odds ratios are adjusted for age, sex, SES and site type, with household random effects.

*n/N*: number of youth who have attained the education outcome/total number of youth included in each analysis (**NB**: *N* differs for each analysis due to different response rates for the questions on the vulnerability categories).

a No children in comparison group who do not attend school regularly.

## Discussion

Our analysis did not find evidence that HIV status affects children's education outcomes in eastern Zimbabwe. The lack of effect of HIV status on education outcomes in younger children could be because HIV-positive children who have survived to primary school age have experienced slower disease progression, have exhibited few signs of infection and are unaware that they are infected. Bandason *et al*. found an association between HIV status and being in the correct grade-for-age in primary school children in Harare, which was attributable to illness (Bandason et al., 2013). However, they did not adjust for potential confounders, such as orphanhood, a possible alternative explanation for poorer educational outcomes, as is suggested in our analysis. Associations between HIV and education outcomes amongst adolescents might have been expected considering other evidence that HIV-positive adolescents experience AIDS-related illnesses (Eaton et al., 2013; Ferrand et al., 2010) which could impact negatively on school attendance and performance. We saw no evidence of this, perhaps due to the small number of HIV-positive children of school-going age in our sample. If sick children and adolescents were less likely to participate in the survey, our results could be subject to selection bias. However, only 0.65% (5/768) of non-respondents in our child survey were due to illness.

Although a child's HIV infection status did not affect their education outcomes, being a young-carer and orphanhood had negative impacts on education outcomes. Between 2009 and 2011, young carers were significantly less likely to attend school regularly than their unaffected peers. As Cluver describes, this is likely due to the time constraints and high burden placed on children who must care for ailing relatives or younger siblings after a parent has died (Cluver, Operario, Lane, & Kganakga, 2012).

Orphanhood significantly reduced the odds of children being in the correct grade for their age and completing primary school. Previous studies, summarised in a review by Guo *et al*. also found orphaned children to be educationally disadvantaged compared to their peers (Guo et al., 2012). Most commonly, studies reported that orphaned children were less likely to be enrolled in school or to attend regularly (Case et al., 2004; Evans & Miguel, 2007; Monasch & Boerma, 2004). However, other studies (Birdthistle et al., 2009; Kürzinger et al., 2008; Oladokun, Brown, Aiyetan, Ayodele, & Osinusi, 2009; Sharma, 2006) found, as we did, that orphanhood had no effect on enrolment or attendance. In addition to the effects of orphanhood varying by country, due to differences in potential cofactors (Guo et al., 2012), the lack of effect we saw could also have been due to orphaned children being relocated to live with more well-off relatives, who were willing and able to provide for their education. Although the numbers were low, we found that orphaned children living in households who reported receiving external support were more likely to be in the correct age-for-grade than those not receiving support. External support received by vulnerable households could have permitted families to send their children to school on a regular basis. This possibility is supported by recent findings from our study population that both conditional and unconditional cash transfers to households increased school attendance amongst vulnerable children (Robertson et al., 2013); findings which echo those from other settings (Kremer, Brannen, & Glennerster, 2013).

The few studies that have examined the gap in schooling found, as we did, that children who lost either or both of their parents to AIDS were less likely to be in the correct grade for their age due to the interruption in studies caused by parental illness preceding death (Ainsworth et al., 2005; Bicego, Rutstein, & Johnson, 2003). Some of these studies have found that it is not atypical for households where one or more parents have died of AIDS to prioritise the enrolment of older children while delaying enrolling the younger ones (Guo et al., 2012).

In a previous study using data from the first two rounds of the survey in Manicaland, all types of orphans were less likely to have completed primary school than their unaffected counterparts (Nyamukapa & Gregson, 2005). Other studies have found that orphanhood can hinder general educational attainment due to the interruption of parental illness and death (Ainsworth et al., 2005; Bicego et al., 2003). Moreover, children orphaned by AIDS have been reported to be less confident and more impulsive, anxious and aggressive in school than other children (Tu et al., 2009). This disruptive behaviour, along with the potential psycho-social impacts of AIDS orphanhood (Nyamukapa et al., 2008, 2010), could account for the poor school performance of orphaned children.

We found that the percentage of children behind in school in Manicaland (from 2009 to 2011) increases from 17.4% (95% CI: 15.8–19.0%) overall to 23.1% (95% CI: 20.2–26.0%) for paternal orphans and 25.4% (95% CI: 19.7–31.1%) for maternal orphans. This is particularly worrisome given the continued high levels of orphanhood in Zimbabwe (Zimbabwe Ministry of Health and Child Welfare, 2012). The lower education levels experienced by orphans compared to other children have potentially severe negative consequences for childhood development and life outcomes considering that children who miss out on education often struggle as adults to find employment, and, when they do find a job, typically earn less than their educated counterparts (Plan, 2008). Exacerbating this problem is the economic situation of Zimbabwe. Initially, after Independence in 1980, Zimbabwe's primary education was free of charge, but fees were introduced in 1991 (Kanyongo, 2005). Fees have risen steadily since then, and, with the economic crash in the last 2000s, many families, particularly in rural areas, were either unable to afford school fees, or needed their children to help out at home earning money. For children who grow up in rural areas, such as those in Manicaland, education enables them to make better use of the resources available, whether by teaching them how to increase the productivity of their land, how best to sell excess produce and how to manage their household budget (Plan, 2008). Without the skills that proper education imparts, children already disadvantaged by the loss of a parent may continue to fall further behind their more educated peers.

## Conclusions

Whilst we found no evidence that HIV-infected children suffered poorer educational outcomes in this population, our findings add to the body of knowledge which suggests that children from families impacted by the HIV epidemic, particularly orphaned children, are less likely to succeed in school than children less affected by AIDS. Orphaned children were more likely than non-vulnerable children to live in a household receiving external support and those that did had improved education outcomes, although the coverage of these programmes was modest in the study populations. Recent studies have shown that cash transfer programmes, in particular, can be an effective means of promoting education (Kremer et al., 2013; Robertson et al., 2013). However, for schools to be able to support children made vulnerable by HIV/AIDS, it is necessary that they remain accessible to as many children as possible, which can be a challenge due to the many barriers that exist at the individual and institutional levels (Schenker & Nyirenda, 2002). Therefore, a combination approach that provides incentives for children to attend school and also equips schools with the tools to support vulnerable children may be most effective in improving education outcomes.
